# *Paragonimiasis* presenting as pyopneumothorax with direct visualization of live worms via video-assisted thoracoscopic surgery: case report

**DOI:** 10.3389/fmed.2026.1928012

**Published:** 2026-08-05

**Authors:** Xingshu Zhang, Ming Gong, Yanping Tang, Jian Li

**Affiliations:** 1 Department of Thoracic Surgery, First Affiliated Hospital of Guizhou University of Traditional Medicine, Guiyang, China; 2 Department of Thoracic Surgery, The Affiliated Hospital of Zunyi Medical University, Zunyi, China

**Keywords:** case report, live worm, *Paragonimiasis*, pyopneumothorax, video-assisted thoracoscopic surgery (VATS)

## Abstract

**Background:**

*Paragonimiasis* is a food-borne zoonotic parasitic disease caused by *Paragonimus* species, mainly acquired through ingestion of raw or undercooked freshwater crustaceans. Owing to its non-specific clinical manifestations, this disease is often misdiagnosed as pulmonary tuberculosis, bacterial pneumonia, or lung cancer.

**Case presentation:**

The patient was a 54-year-old male who initially presented with pyopneumothorax, along with a history of raw food consumption, chest wall subcutaneous nodules, peripheral eosinophilia, and left-sided pyopneumothorax. Video-assisted thoracoscopic surgery (VATS) was performed, during which live adult *Paragonimus* worms were directly visualized and extracted; post-operatively, the patient received praziquantel for antiparasitic treatment. He achieved complete recovery following VATS debridement combined with praziquantel therapy.

**Conclusion:**

This case underscores the diagnostic challenges posed by atypical pleural *Paragonimiasis* and highlights that VATS has important dual diagnostic and therapeutic value for highly suspected cases with inconclusive non-invasive workup in endemic regions, while serological testing and parasite ova detection remain first-line confirmatory methods for typical cases.

## 1 Introduction

*Paragonimiasis*, also referred to as distomatosis, is a chronic food-borne zoonotic disease caused by parasites of the genus *Paragonimus*. Infection mainly results from ingestion of raw or undercooked freshwater crustaceans (crabs and crayfish) (1), and this disease is endemic in many parts of the world, particularly in Asia (2, 3). Although it has a high incidence in certain regions, its diverse and non-specific clinical manifestations often lead to misdiagnosis as pulmonary tuberculosis, bacterial pneumonia, or even lung cancer, resulting in delayed diagnosis and treatment (4, 5). We herein report a rare case of *Paragonimiasis* that initially presented with pyopneumothorax, which was definitively diagnosed by direct visualization of live adult worms during thoracoscopy and confirmed by pathological biopsy. We also summarize the parasite life cycle, clinical staging, diagnostic clues, and management strategies for atypical *Paragonimiasis* based on a literature review.

## 2 Case presentation

**Figure 1:**
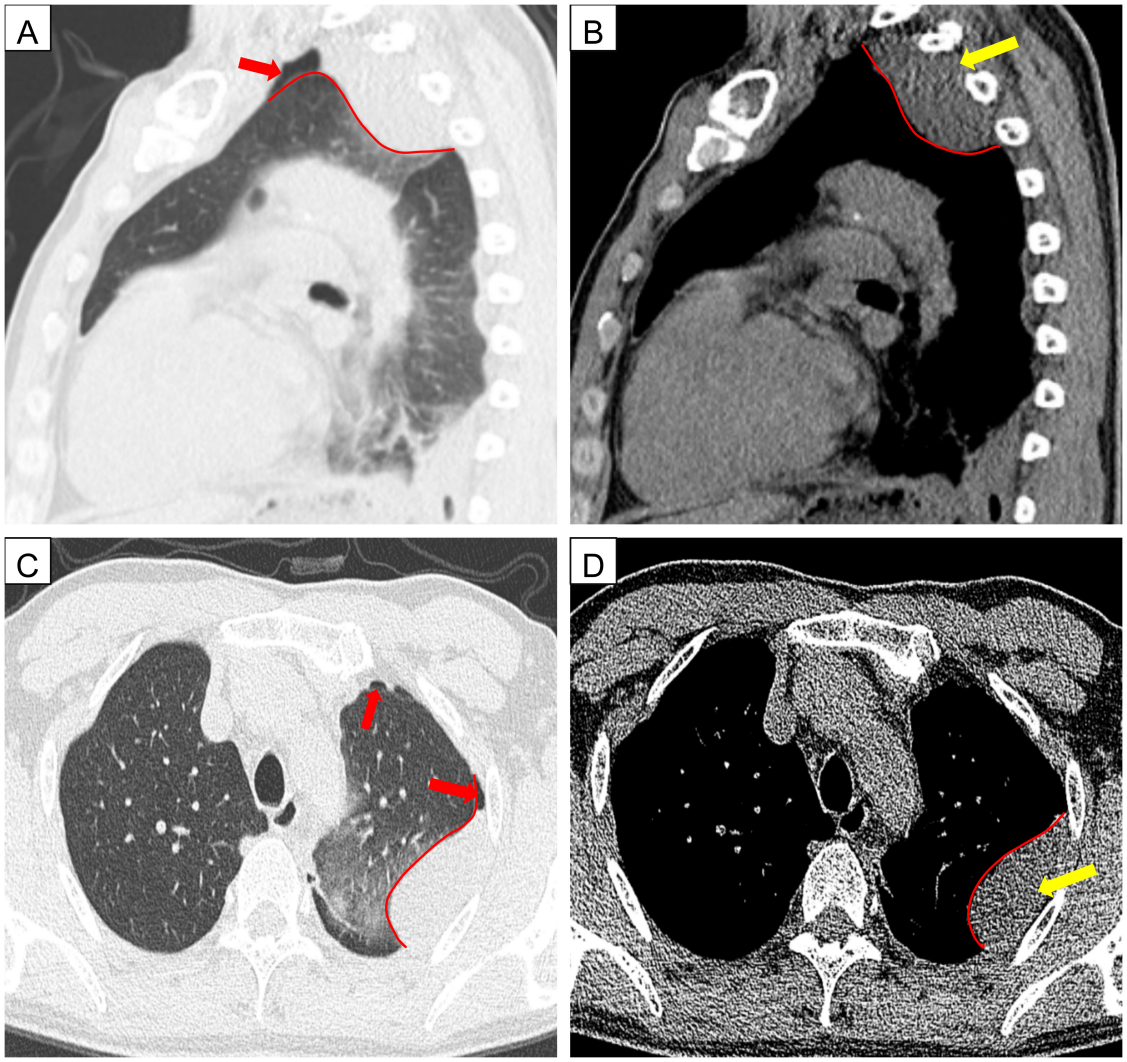
Admission chest CT showed loculated hydropneumothorax, which was further confirmed as pyopneumothorax intraoperatively. **(A, B)** Sagittal images; **(C, D)** Axial images. Red solid arrows indicate loculated pneumothorax, yellow arrows indicate pleural effusion, and the red solid line indicates the border of compressive atelectasis of the left lung.

A 54-year-old male was admitted to the Department of Thoracic Surgery for exertional chest tightness, dyspnea, and left chest pain lasting more than 1 month, with acute aggravation in the past 2 days. He developed the above symptoms without obvious pre-disposing factors, accompanied by an occasional cough with scant white mucoid sputum. There was no fetid purulent sputum, hemoptysis, fever, night sweats, palpitations, or tachycardia. Symptomatic treatment at a clinic for 1 week (specific medications unknown) showed no improvement. His condition deteriorated markedly 2 days before admission with severe exertional dyspnea. Chest computed tomography (CT) revealed scattered streaky opacities in the left lung suggestive of inflammation, left hydropneumothorax (approximately 10% lung compression) involving the interlobar fissure, and compressive atelectasis of the left lung (Figure 1). On admission, vital signs were stable. Two painless subcutaneous nodules (maximum diameter 0.6 to 0.8 cm) were palpable on the left costal margin. Breath sounds were decreased in the left lung, and crackles were heard in the left lower lung zone.

Laboratory results on admission are summarized in Table 1. Sputum bacterial culture and a routine stool test for parasite ova were negative. Sputum acid-fast bacilli (AFB) smear was negative, and the T-SPOT. TB test was negative. Routine biochemical parameters and myocardial enzyme profile were normal. Electrocardiography showed a sinus rhythm.

**Table 1:** Admission peripheral blood complete blood count with differential.

Parameter	Result	Reference range
White blood cell count	6.41 × 10^9^/L	3.5–9.5 × 10^9^/L
Neutrophil percentage	51.6%	40%-75%
Lymphocyte percentage	29.0%	20%-50%
Monocyte percentage	5.3%	3%-10%
Eosinophil percentage	14.1%	0.4%-8.0%
Absolute eosinophil count	0.90 × 10^9^/L	0.02–0.52 × 10^9^/L
Hemoglobin	132 g/L	120–160 g/L
Platelet count	254 × 10^9^/L	100–300 × 10^9^/L

**Figure 2:**
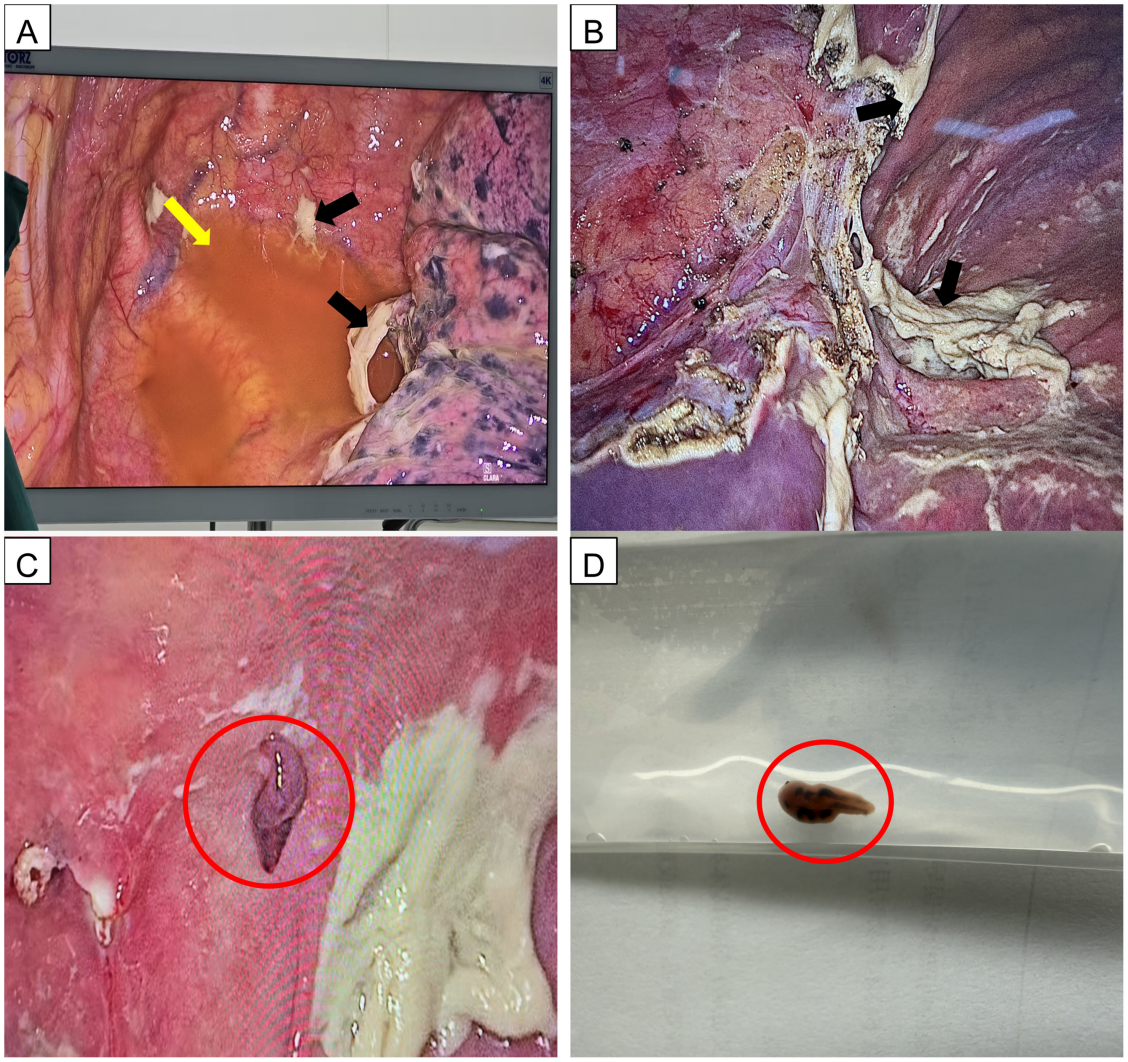
Intraoperative thoracoscopic findings and parasite specimen. **(A, B)** Yellow purulent effusion (yellow arrows) and local pleural adhesions are observed in the left thoracic cavity, with purulent exudate on the lung surface and chest wall (black arrows). **(C)** The thoracoscopic view shows a wriggling live adult *Paragonimus* on the chest wall (red circle). **(D)** Gross specimen of the removed live worm (red circles).

After consultation with the Department of Respiratory Medicine, a pulmonary infection complicated by left pleural effusion was diagnosed. The patient was treated with cefoperazone-sulbactam sodium, and pre-operative preparation for thoracoscopic debridement of empyema was completed. Intraoperatively, approximately 500 ml of yellow purulent pleural effusion and local pleural adhesions were found in the left thoracic cavity (Figures 2A and B). Pleural fluid samples were collected for routine, biochemical, and bacterial culture analyses, followed by complete evacuation of the pus. A small portion of the purulent fibrous plate was harvested for pathological examination. Further exploration detected a wriggling live adult worm on the lateral chest wall (Figures 2C and D), which was removed and submitted for biopsy. Pleural fluid analysis results are detailed in Table 2.

**Table 2:** Pleural fluid analysis results.

Category	Parameter	Result	Reference range
General appearance	Appearance	Yellow turbid fluid	–
	Rivalta test	Positive	–
	Spontaneous coagulum	Present	Absent (transudate); present (exudate)
Routine cytology	Total white blood cell count	17,426 × 10^6^/L	< 500 × 10^6^/L (transudate); >1,000 × 10^6^/L (exudate)
	Polymorphonuclear cell percentage	3.9%	–
	Mononuclear cell percentage	96.1%	–
	Eosinophil differential count	Not performed	–
Biochemical parameters	Total protein	77.16 g/L	< 30 g/L (transudate); >35 g/L (exudate)
	Glucose	0.09 mmol/L	3.33–5.55mmol/L (transudate); < 3.33mmol/L (exudate)
	Lactate dehydrogenase (LDH)	214.32 U/L	< 200 U/L (transudate); >200 U/L (exudate)
	Adenosine deaminase	28 U/L	0–20 U/L
Microbiological tests	Gram stain	No bacteria detected	–
	Aerobic/anaerobic bacterial culture	Negative	–
	Acid-fast bacilli smear	Negative	–
	Mycobacterium tuberculosis PCR	Negative	–

**Figure 3:**
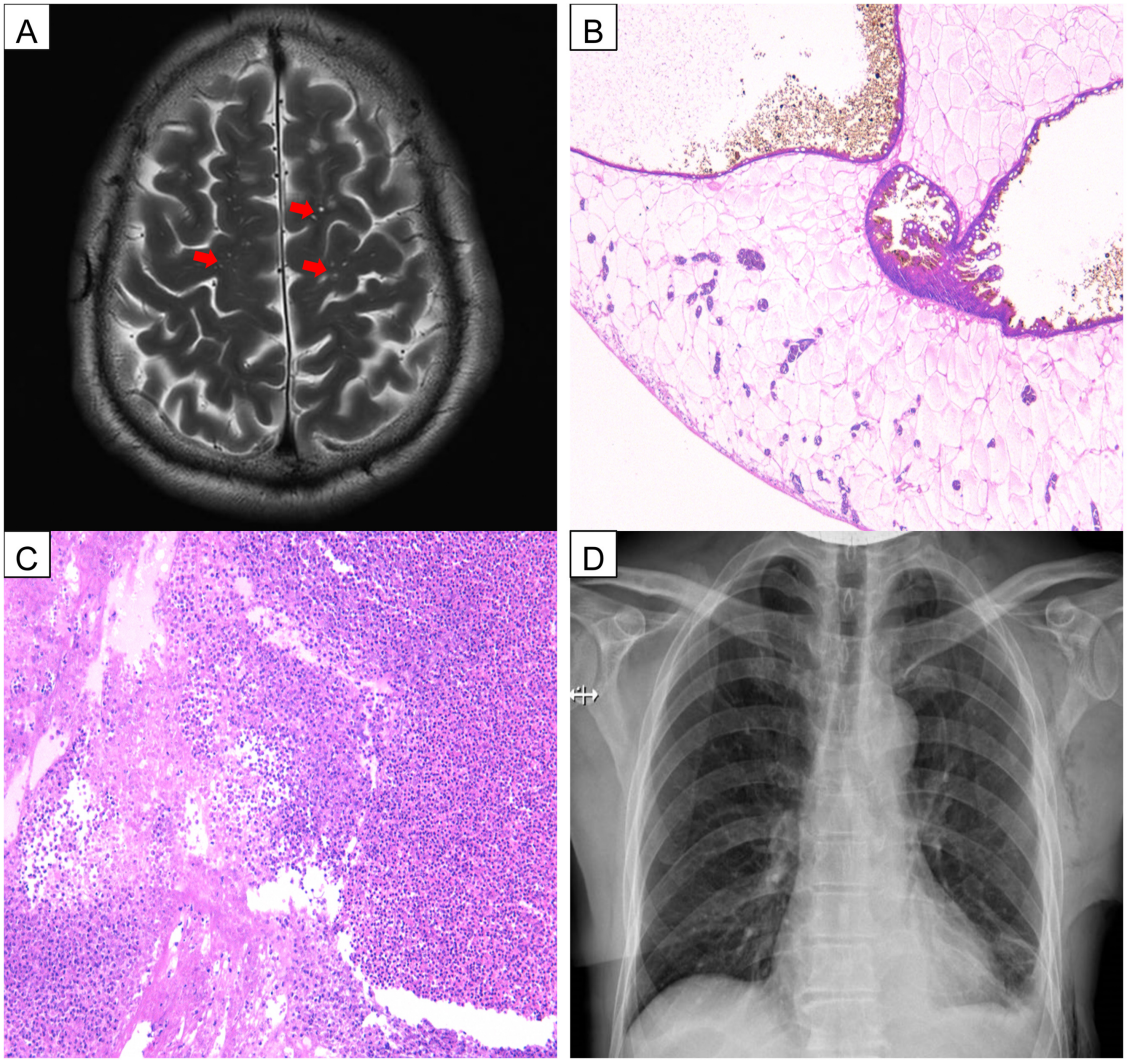
Cranial imaging, pathological findings, and post-operative follow-up. **(A)** Cranial MRI (FLAIR sequence) showing multiple scattered punctate hyperintense lesions in the periventricular and centrum semiovale white matter (red arrows). The etiology of these lesions was non-specific, and ectopic *Paragonimiasis* involvement could not be completely excluded. **(B)** Gross and microscopic features of the extracted parasite are consistent with *Paragonimus* species. **(C)** Histopathological image of the pleural fibrous peel showing suppurative inflammation with extensive necrosis. **(D)** Post-operative chest radiograph showing satisfactory re-expansion of the left lung, with no residual significant effusion or pneumothorax.

The pleural fluid/serum total protein ratio was 1.14, which exceeded the 0.5 threshold of Light's criteria, confirming the fluid as an exudate. Concurrent serum biochemical parameters for Light's criteria calculation are shown in Table 3. Post-operative pathology confirmed that the thoracic specimen was a parasite consistent with *Paragonimus* species. Grossly, the worm was approximately 8 mm in length and 4 mm in width, oval-shaped, reddish-brown, with a flat ventral surface and a slightly convex dorsal surface; the oral sucker and ventral sucker were visible on the ventral side. Microscopically, the body wall was thick with distributed cuticular spines, and internal organ structures were identifiable, consistent with the morphological features of an adult *Paragonimus* worm. The fibrous plate showed suppurative inflammation with massive necrosis (Figures 3B and C). Further workup after infectious disease consultation showed a negative stool examination for parasite eggs. Cranial magnetic resonance imaging (MRI) demonstrated multiple scattered punctate slightly long T1 and long T2 signals in the cerebral white matter around the bilateral centrum semiovale and lateral ventricles, with hyperintensity on FLAIR and no obvious hyperintensity on diffusion-weighted imaging (DWI) (Figure 3A). These imaging findings were non-specific; ectopic *Paragonimus* involvement could not be completely excluded, and alternative etiologies such as chronic ischemic white matter changes were also considered. The patient had no neurological symptoms such as headache, vomiting, or limb dysfunction on admission. After multidisciplinary consultation with neurology and infectious disease specialists, lumbar puncture for cerebrospinal fluid examination was not performed. The justifications for this decision were as follows: (1) the patient had no neurological symptoms or positive signs; (2) the cranial MRI showed only non-specific white matter changes without typical features of cerebral *Paragonimiasis*; (3) the yield of cerebrospinal fluid examination was considered low for asymptomatic patients; (4) the patient refused the invasive procedure. Close follow-up with serial cranial imaging and neurological assessment was planned instead. The diagnosis of *Paragonimus* infection was confirmed by chest wall subcutaneous masses, peripheral eosinophilia, direct visualization of a live adult worm in the thoracic cavity during surgery, and pathological verification. Detailed history revealed the patient had long lived in an ethnic minority area of RongJiang County, Qiandongnan Prefecture, Guizhou Province, China, and had a habit of eating raw beef, sashimi, crabs, and other raw foods.

**Table 3:** Concurrent serum biochemical parameters for Light's criteria calculation.

Parameter	Result	Reference range
Total protein	67.8 g/L	60–80 g/L
Albumin	47 g/L	35–55 g/L
LDH	188 U/L	120–250 U/L

The pleural fluid/serum total protein ratio was calculated as 1.14 (>0.5 threshold), confirming the effusion as an exudate according to Light's criteria.

The patient underwent thoracoscopic debridement and drainage of empyema and was discharged on post-operative day 7. By that time, the left pyopneumothorax had resolved, and the left lung re-expanded well (Figure 3D). Per the recommendation of the Department of Infectious Diseases, he was treated with praziquantel (body weight 72 kg) 1,800 mg PO tid, 3 days per course, with a 7-day interval between courses, for a total of 3 deworming courses.

**Figure 4:**
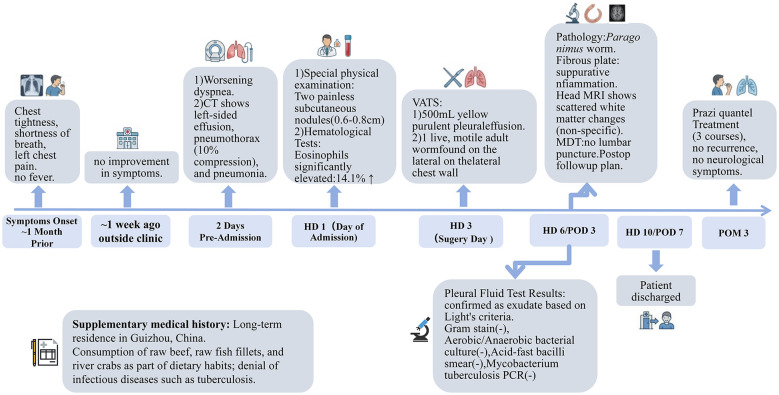
Timeline of the patient's course.

This regimen is consistent with the standard dose of 75 mg/kg per day for *Paragonimiasis* treatment recommended by Chinese clinical guidelines for parasitic diseases (6). At the 3-month follow-up after treatment, the chest wall subcutaneous nodules disappeared, the eosinophil count decreased significantly to 0.50 × 10^9^/L, and the patient remained in good general condition with no neurological symptoms. The complete diagnostic and therapeutic process is summarized in Figure 4.

## 3 Discussion

### 3.1 Pathogenic mechanism of pleural lesions in *Paragonimiasis*

*Paragonimiasis* is a chronic foodborne zoonotic parasitic disease caused by trematodes of the genus *Paragonimus*. Its global epidemiological distribution is closely linked to dietary practices of consuming raw or undercooked freshwater crustaceans (1, 2). Clarifying the parasite's life cycle and pathogenic process in humans is critical for interpreting the clinical manifestations and complications observed in this case.

Human infection is acquired via ingestion of raw or undercooked crabs or crayfish harboring infective *Paragonimus* metacercariae (1, 2). Following ingestion, metacercariae excyst in the duodenum under the action of digestive juices, releasing immature larvae with strong tissue-invasive capacity. The larvae penetrate the intestinal wall to enter the peritoneal cavity, migrate along the surface of abdominal viscera, and subsequently traverse the diaphragm and pleura to reach the pleural cavity and eventually the lung parenchyma (7–9). Mechanical damage and metabolic antigens released during larval migration trigger intense inflammatory and allergic reactions in the pleura, leading to pleuritic chest pain, exudative pleural effusion, and pleural adhesion (7). Direct penetration of the visceral pleura by migrating larvae causes pneumothorax; necrotic inflammatory exudates further result in pyopneumothorax and dyspnea (10, 11), which is consistent with the initial presentation of our patient. The purulent appearance of the effusion is mainly caused by severe inflammatory necrosis induced by parasite antigens, rather than primary bacterial infection, as evidenced by negative bacterial cultures.

After reaching the lung parenchyma, larvae mature into adult worms over 5–6 weeks, become encapsulated by fibrous cysts, and begin oviposition (12). Eggs are either expectorated in sputum or swallowed and excreted in feces, corresponding to classic chronic symptoms of established infection such as chronic cough, rusty sputum, and hemoptysis (9). Ectopic migration of larvae to subcutaneous tissue, the brain, or abdominal organs causes extrapulmonary *Paragonimiasis* (13–16), which accounts for the chest wall subcutaneous nodules and intracranial imaging abnormalities observed in this case.

### 3.2 Clinical staging and manifestation analysis of this case

In the early stage of *Paragonimiasis* (within 2 months of infection), the disease is characterized by larval migration and allergic-inflammatory responses. Common manifestations include chest pain, pleural effusion, pneumothorax, migratory subcutaneous nodules, and marked peripheral eosinophilia. Because the parasites have not yet reached maturity, sputum and stool examinations for ova are usually negative, which pre-disposes to misdiagnosis; imaging findings are often non-specific, showing migratory infiltrates, effusion, or pneumothorax (9, 17). Our patient, with a disease course of 1 month, presented with pyopneumothorax and subcutaneous nodules, had negative egg detection, and showed marked eosinophilia, all consistent with early-stage infection. In contrast, in the late stage (after 2 months of infection), adult worms colonize the lung parenchyma and form encapsulated cysts. Clinically, patients typically present with chronic cough, rusty sputum, recurrent hemoptysis, and intermittent chest pain. The egg detection rate in sputum or stool is relatively high, and imaging reveals nodules, cystic lesions, cavities, and pleural thickening (9, 17).

### 3.3 Diagnostic dilemma and differential diagnosis strategy

The diagnosis of pleural *Paragonimiasis* remains challenging due to the lack of specificity in both clinical and imaging findings. In this case, the patient presented with pyopneumothorax and purulent pleural effusion and was initially suspected of having bacterial empyema. However, three key clinical clues strongly suggested a parasitic infection: a history of consuming raw freshwater crustaceans in an endemic area, subcutaneous nodules on the chest wall, and markedly elevated peripheral blood eosinophilia.

For exudative pleural effusion accompanied by eosinophilia, the differential diagnosis should include tuberculous pleuritis, bacterial empyema, malignant pleural effusion, and parasitic infections such as echinococcosis and sparganosis (18). In this case, the pleural fluid profile-an exudate with low glucose and elevated adenosine deaminase (ADA)-overlapped with that of tuberculous pleuritis, but negative acid-fast bacilli smears, a negative pleural fluid Mycobacterium tuberculosis PCR, and a negative T-SPOT. TB assay collectively ruled out active tuberculous infection, and peripheral eosinophilia is also rare in typical tuberculous pleurisy. Although the pleural fluid appeared purulent, Gram stain and both aerobic and anaerobic bacterial cultures were negative, which did not support primary bacterial empyema. In a middle-aged patient with unilateral exudative pleural effusion, malignant effusion secondary to lung cancer must be routinely considered; here, chest CT revealed no definite space-occupying lesion in the lung parenchyma, pleural fluid cytology detected no malignant cells, and post-operative pathological examination of the pleural fibrous peel showed only suppurative inflammation with no malignant cells, and the definitive identification of a *Paragonimus* worm ultimately excluded the diagnosis of lung cancer.

Conventional confirmatory diagnostic methods rely on detection of ova in sputum or stool, serological antibody testing, or histopathological confirmation. However, in the early stage of infection, due to the pre-patent period before worm maturity and low worm burden, the false-negative rate of ova detection is high (19). Serological assays such as enzyme-linked immunosorbent assay (ELISA) offer high sensitivity and are of significant diagnostic value in early cases (20).

Regarding species identification, molecular biological confirmation was not performed in this case. Based on the epidemiological characteristics of the Qiandongnan Miao and Dong Autonomous Prefecture in Guizhou Province of China, *Paragonimus westermani* is the pre-dominant endemic species in this region. Combined with the morphological features of the adult worm specimen, the pathogen is highly suspected to be *P.westermani. Paragonimus skrjabini*, another endemic species in southwestern China, rarely develops into adult worms in humans and typically manifests pre-dominantly as cutaneous larva migrans (21, 22).

Concerning central nervous system (CNS) involvement, given the patient's systemic infectious status and abnormal cranial MRI findings, ectopic cerebral *Paragonimus* involvement could not be completely excluded, but the imaging findings were non-specific, and other etiologies could not be ruled out. However, because the patient had no neurological symptoms, a cerebrospinal fluid examination was not performed. The patient was treated with a standard dose of praziquantel, and regular neurological evaluation and imaging follow-up are planned.

### 3.4 Imaging features and diagnostic-therapeutic value of VATS in atypical pleural *Paragonimiasis*

Chest imaging manifestations of pleural *Paragonimiasis* are highly heterogeneous, presenting as pulmonary nodules, cystic lesions, cavitations, pleural thickening, pleural effusion, pneumothorax, and other signs (10, 11, 23). Chest CT in this case showed scattered streaky opacities in the left lung, interlobar loculated effusion, and hydropneumothorax, indicating concurrent involvement of the lung parenchyma and pleura.

Previous studies have confirmed that the “tunnel sign” formed by larval migration within lung parenchyma is a highly suggestive imaging feature that assists diagnosis when combined with epidemiological history and eosinophilia (24–26). No definitive tunnel sign was observed in this patient, presumably related to lesion masking by compressive atelectasis secondary to pneumothorax and pleural effusion. Clinically, it should be noted that pneumothorax or hydropneumothorax can be the initial presentation of the disease, and *Paragonimiasis* should be included in the differential diagnosis for unexplained pneumothorax in endemic areas.

For cases with atypical imaging features and diagnostic difficulties, VATS bears dual core diagnostic and therapeutic values (27). Diagnostically, VATS allows direct exploration of the pleural cavity and detection of live worms; direct visualization combined with histopathological examination serves as a definitive diagnostic approach superior to indirect laboratory tests. Therapeutically, it enables complete removal of purulent effusion and fibrous peel to effectively control infection and promote lung re-expansion. VATS provides effective local source control of pleural lesions, and subsequent systemic praziquantel therapy is required for radical cure of the parasitic infection; the combined strategy achieves favorable clinical outcomes.

Several limitations of this study should be acknowledged. First, species-level identification was not performed via molecular biology or specialist parasitological examination, and the inference of *P. westermani* was based only on regional epidemiology and basic morphological features, which may have potential bias. Second, eosinophil differential count in pleural fluid was not available, which would have provided additional supportive evidence for the diagnosis. Third, cerebrospinal fluid examination was not performed to confirm cerebral involvement due to the absence of neurological indications.

## 4 Conclusion

This case illustrates the diagnostic complexity of atypical pleural *Paragonimiasis*, particularly in the early stage of infection. In endemic regions, *Paragonimiasis* should be included in the differential diagnosis for patients with unexplained pleural disease, especially when accompanied by eosinophilia and a history of consuming raw freshwater crustaceans. For highly suspected cases with inconclusive non-invasive examinations, early thoracoscopic exploration can provide definitive diagnosis and effective local source control, and radical cure can be achieved with subsequent standard praziquantel therapy.

## Data Availability

The original contributions presented in the study are included in the article/Supplementary material, further inquiries can be directed to the corresponding author.
